# Crosstalk between Environmental Inflammatory Stimuli and Non-Coding RNA in Cancer Occurrence and Development

**DOI:** 10.3390/cancers13174436

**Published:** 2021-09-02

**Authors:** Tingting Xu, Mengyan Xie, Xinming Jing, Jiahua Cui, Xi Wu, Yongqian Shu

**Affiliations:** 1 Department of Oncology, The First Affiliated Hospital of Nanjing Medical University, Nanjing 210003, China; xttiris96@163.com (T.X.); mengyan_xie@outlook.com (M.X.); jingxinmingNJMU@163.com (X.J.); cjh_njmu@126.com (J.C.); wuxiolivia@126.com (X.W.); 2 Department of Oncology, Sir Run Run Hospital, Nanjing Medical University, Nanjing 210003, China; 3 Jiangsu Key Lab of Cancer Biomarkers, Prevention and Treatment, Collaborative Innovation Center for Cancer Personalized Medicine, Nanjing Medical University, Nanjing 210003, China

**Keywords:** ncRNA, miRNA, lncRNA, circRNA, inflammation, cancer, environment

## Abstract

**Simple Summary:**

Increasing evidence has indicated that chronic inflammatory processes have an influence on tumor occurrence and all stages of tumor development. A dramatic increase of studies into non-coding RNAs (ncRNAs) biology has shown that ncRNAs act as oncogenic drivers and tumor suppressors in various inflammation-induced cancers. Thus, this complex network of inflammation-associated cancers and ncRNAs offers targets for prevention from the malignant transformation from inflammation and treatment of malignant diseases.

**Abstract:**

There is a clear relationship between inflammatory response and different stages of tumor development. Common inflammation-related carcinogens include viruses, bacteria, and environmental mutagens, such as air pollutants, toxic metals, and ultraviolet light. The expression pattern of ncRNA changes in a variety of disease conditions, including inflammation and cancer. Non-coding RNAs (ncRNAs) have a causative role in enhancing inflammatory stimulation and evading immune responses, which are particularly important in persistent pathogen infection and inflammation-to-cancer transformation. In this review, we investigated the mechanism of ncRNA expression imbalance in inflammation-related cancers. A better understanding of the function of inflammation-associated ncRNAs may help to reveal the potential of ncRNAs as a new therapeutic strategy.

## 1. Introduction

In 1863, Rudolf Virchow discovered white blood cells in tumor tissues, suggesting a link between inflammation and cancer. Later on, it was discovered that cancer most often occurs at the site of chronic inflammation [1]. Individuals with chronic inflammation, regardless of infectious agents, are susceptible to various cancers. This phenomenon was observed in patients with gastric cancer (GC), hepatocellular carcinoma (HCC), and colorectal cancer (CRC) [2,3]. Cancer cells and a complex network of stromal cells that are comprised of innate cells (monocytes, macrophages, neutrophils, mast cells, dendritic cells, natural killer (NK) cells, and others), adoptive immune cells (T cells and B cells), and myeloid and lymphoid lineages together form the tumor microenvironment (TME). It is now evident that location and proportions or activation states of different immune cell subsets vary between different tumor types and also between individuals with same cancers [4]. Chronic inflammation predisposes to tumorigenesis or the development of cancers by suppressing anti-tumor immunity and directly influencing pro-tumorigenic signals and functions.

**Figure 1:**
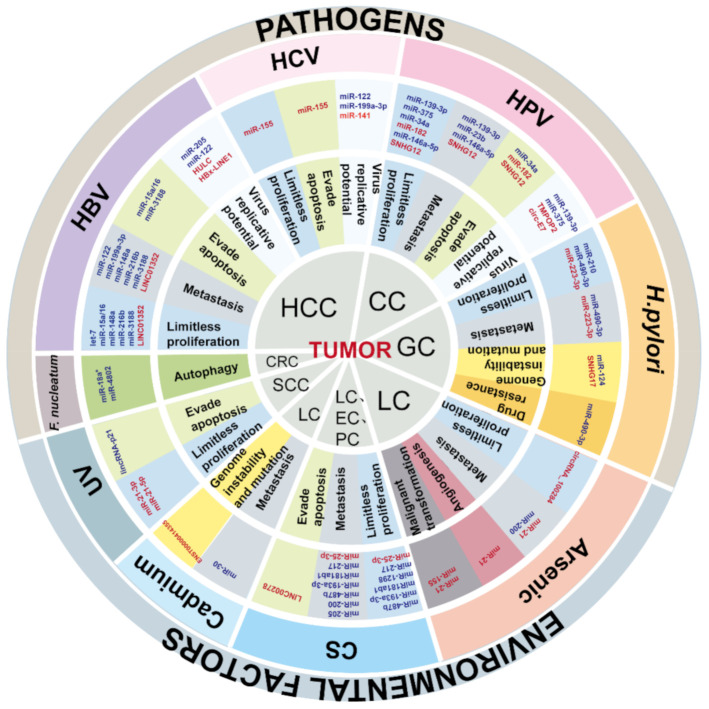
The schematic summation of ncRNA dysregulation and relevant cancer-promoting mechanisms. The ncRNAs up-regulated and down-regulated are depicted in red and blue, respectively.

“The central dogma of biology”, enunciated by Francis Crick in 1958, regarded RNA as the intermediate between DNA and proteins [5]. In 1993, Lee et al. discovered the first small non-coding RNA (ncRNA) lin-4 in *Caenorhabditis elegans* by genetic analysis [6], and then ncRNAs were brought into the research field. Later, advances in sequencing technology led to the discovery of a large number of ncRNA species. As depicted in Figure 1, plenty of studies have proposed that aberrant ncRNAs induced by inflammation may lead to tumorigenesis and influence cancer development. Non-coding RNAs, which account for 98% of the RNA [7], are classified into long ncRNAs (lncRNAs), microRNAs (miRNAs), small nucleolar RNAs (snoRNAs), and circular RNAs (circRNAs) [8]. Non-coding RNAs longer than 200 nucleotides (nts) are defined as lncRNAs, and lncRNAs and mRNAs share many common features, such as transcription, splicing, capping, and polyadenylation by RNA polymerase II-like protein-coding pathways [9]. MicroRNAs, which are perhaps the most extensive-studied short ncRNAs, are approximately 23 nts in length. MicroRNAs usually seek target mRNAs that have complementarity to a seed region in the 3′-untranslated region (UTR) or 5′-UTR of them via RNA-induced silencing complex (RISC). The binding of miRNAs and mRNAs leads to the degradation of target mRNAs or prevents them from being translated into proteins [10]. Small nuclear RNAs are mainly distributed in the nucleolus of eukaryotic cells and are related to the processing and modification of rRNAs. Additionally, circRNAs, characterized by a covalently closed-loop structure, are also highly representative ncRNAs in the eukaryotic transcriptome.

## 2. Aberrant Regulation of ncRNAs under Inflammation Stimuli

### 2.1. Transcriptional Regulation

#### 2.1.1. Epigenetic Modification

**Figure 2:**
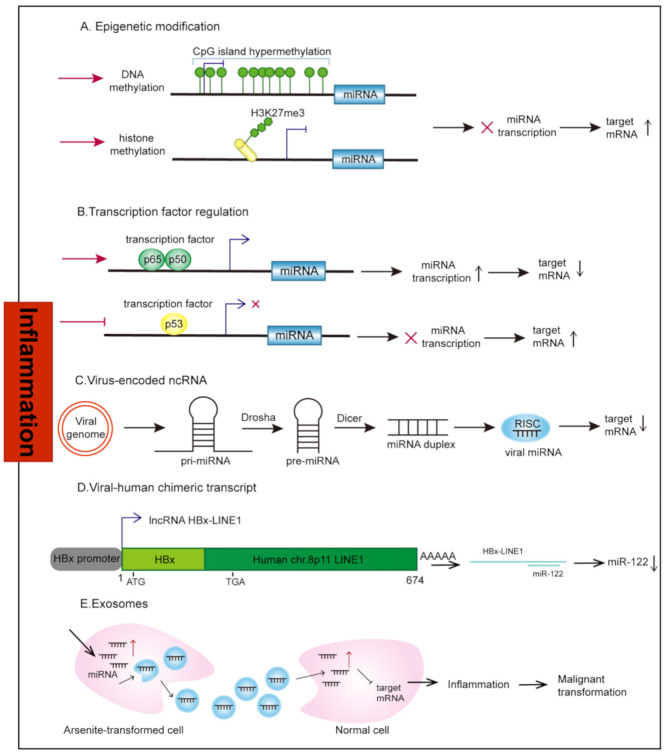
Mechanisms of ncRNA dysregulation under inflammation. (**A**) The alteration in the epigenetic modifications of ncRNAs is involved in ncRNA dysregulation under inflammation. Specifically, CpG island hypermethylation or histone methylation-associated silencing of miRNAs causes an upregulation of their target mRNAs. (**B**) TFs bind to the promoter of ncRNAs to transcriptionally regulate their expression. (**C**) Viral genome with DNA components uses the host’s biochemical mechanism to encode viral ncRNAs. (**D**) HBx-LINE1, which is transcribed by the viral HBX promoter, can serve as a molecular sponge for sequestering miR-122. (**E**) Exosome-derived miRNAs from arsenite-transformed cells are transferred into normal cells via exosomes to increase miRNA levels in uninfected cells and induce arsenite-induced malignant transformation.

The epigenetic modifications inhibit or activate transcription of ncRNAs by adding or removing chemical groups such as methyl-(${\mathrm{CH}}_{3}$), phosphor-(${\mathrm{PO}}_{3}$), and acetyl-(${\mathrm{CH}}_{3}\mathrm{CO}$) groups to DNA, chromatin, and histones and by modifying the structure and accessibility of DNA (Figure 2A). Among these commonly observed epigenetic alterations, methylation that occurs in cytosine-guanine dinucleotide-rich areas (CpG islands) in gene promoter regions under the catalysis of DNA methyltransferases (DNMTs) is perhaps the most widely studied epigenetic link to inflammation-associated cancers. The best-documented example is that a large number of tumor suppressor ncRNAs are silenced through hypermethylation in *Helicobacter pylori* (*H. pylori*)-infected human gastric mucosa. The ability of *H. pylori* to induce DNA methylation in gastric mucosa remains unclear; increased expression of genes related to inflammatory responses may be a tempting explanation. It is believed that prolonged nuclear factor-kappaB (NF-κB) activation in *H. pylori*-infected mucosa could upregulate DNMTs [11]. Lymphocyte and macrophage infiltration induced by *H. pylori*-mediated inflammation may also have an important role in methylation induction [12]. Moreover, *H. pylori* possess multiple DNMTs and a pili-like structure called the type 4 secretion system (T4SS). Its own DNMTs can be directly injected into epithelial cells to induce gene methylation through T4SS [13].

Other important epigenetic regulators of ncRNA expression under inflammatory stimuli are histone modifications which include acetylation, methylation, phosphorylation, and ubiquitination. So far, most of the reported histone modifications related to inflammation are acetylation/deacetylation and methylation/demethylation. It has been shown that during transforming growth factor-β (TGF-β)-induced epithelial-mesenchymal transition (EMT), repressive histone mark histone 3 lysine 9 dimethylation (H3K9me2), while an increase of activating mark histone H3 lysine 4 tri-methylation (H3K4me3) and H3K36me3 was observed [14]. Moreover, a decreased action of histone deacetylases (HDACs) and an increased action of histone acetyltransferases (HATs) was found under cigarette smoke (CS) exposure [15,16]. CS may induce histone hyperacetylation through influencing the balance of HATs and HDACs, thus regulating ncRNA expression.

#### 2.1.2. Transcription Factors

The inflammatory response is coordinated by a large range of mediators. Among them, transcription factors (TFs) such as NF-κB and Signal Transducer and Activator of Transcription 3 (STAT3) are crucial in mediating inflammation and cancer development [17,18]. During inflammatory stimulation, these TFs bind to the promoter region of ncRNAs to regulate their transcription and expression (Figure 2B). For example, the miR-21 promoter contains a shared NF-κB binding site [19]. In addition, multiple transcriptional regulators can regulate a single ncRNA. For example, not only through regulation by NF-κB, the expression of miR-21 can also be induced by pro-inflammatory cytokine interleukin 6 (IL-6) through the STAT3 [20]. The transcription of ncRNAs is also negatively regulated during inflammatory stimulation. Transcription factor p53 is usually regarded as a major tumor suppressor, and its activation serves to protect against pro-tumorigenic inflammation and oncogenesis [21,22]. Viruses are common inflammation leaders. During virus infection, p53 participates in the protective cellular response to impair effective viral replication. To tackle this, viral-encoded proteins, such as SV40 large T antigen [23], manage to bind and inactivate p53. Non-coding RNAs are important parts of the p53 network and crosstalk with p53 at multiple levels. Members of the tumor-suppressive miR-34 family are the most common p53-activated miRNAs [24]. Inflammatory stimulation and loss of p53 inhibit the expression of related miRNAs. This may be an important step in cancer progression related to inflammation.

### 2.2. Virus-Encoded ncRNAs

The successful survival of viruses depends on their ability to exploit the biosynthetic mechanisms of host cells and inactivate the host’s innate defense mechanisms. The relatively limited viral coding capacity makes tiny ncRNAs particularly effective and accessible tools for shutting down the expression of specific genes to inactivate host cell defenses [25]. In most cases, natural viruses that encode miRNAs have DNA components in their replication cycle. They replicate in the nucleus, where they have full access to the original host miRNAs biogenesis mechanism (Figure 2C). However, not all viruses that meet these conditions encode ncRNAs. For example, the herpesvirus family encodes most known viral miRNAs, except for the varicella-zoster virus [26]. Moreover, there is still controversy as to whether viruses with RNA genomes encode ncRNAs [27,28,29]. Viral lncRNAs are usually transcribed from RNA polymerase II or III. Some viral lncRNAs can even be polyadenylated, similar to host mRNAs [30,31]. With notable exceptions, most viral ncRNAs are low in abundance and lack evolutionary conservation. This means that viral ncRNAs are places for rapid evolution and maybe a driving force for speciation. The reliance on viral ncRNAs rather than viral proteins allows these viruses to escape immune surveillance [32]. An in-depth investigation of truly valuable virus-encoded ncRNAs may reveal the new functions of ncRNAs and their possible clinical relevance.

### 2.3. Human-Virus Fusion ncRNAs

HBx-LINE1 is a chimeric transcript formed by the insertion of the Hepatitis B virus (HBV) gene into the host genome, with a length of 674 bps initiated by the viral HBx promoter (Figure 2D). HBx-LINE1 directly promotes the occurrence of HCC and cell migration through EMT, which is closely related to the poor prognosis of HBV-positive HCC patients [33]. Usually, the insertion site of the HBV gene has a certain relationship with repetitive sequences, such as long scattered repetitive sequences (long interspersed nuclear elements, LINEs), short scattered repetitive sequences (short interspersed nuclear elements, SINEs), or Alu sequence. In the absence of HBV gene fusion, the LINE1 sequence is silent. Unexpectedly high transcriptional activity occurs in the case of HBV gene fusion. HBx-LINE1 chimeric transcript translates a protein composed of 87 amino acids, five of which are formed by LINE1 translation. Yet, this protein has nothing to do with its role in promoting liver cancer. Researchers deliberately modified a terminator in the front portion of the HBX-LINE1 fragment and found that the cancer-promoting effect of the mutant HBX-LINE1 still existed. This indicates that HBx-LINE1 may function through a mechanism similar to the lncRNA. This is significantly different from other classic examples of fusion genes leading to cancer, such as BCR-ABL in leukemia, EML4-ALK in lung cancer (LC), and TMPRSS2-ERG in prostate cancer. Therefore, the discovery of HBx-LINE1 provides new methods and ideas for tumor detection and treatment.

### 2.4. Exosomes

Extracellular vesicles (EVs), which refer to structures restricted by a double layer of lipids, have emerged as new factors in cell–cell communication. All cells in the organism release EVs that are absorbed by nearby cells or circulated in the blood and eventually absorbed by cells at a distance. EVs contain various components of origin cells, which have been proven to facilitate the spread of bacteria as well as viruses from infected to uninfected cells, mainly by masking the antigens and pathogen-associated molecular patterns (PAMPs) of bacterial and viral pathogens, thereby avoiding immunity recognition [34,35,36,37]. Exosomes are nano-sized small EVs derived from endosomes, produced by the fusion of multivesicular bodies and plasma membrane. Exosomes are a specific library that contains mRNAs and ncRNAs. The exosomes containing RNA are released into the extracellular environment and can transmit genetically encoded information to other cells (Figure 2E). Factors that determine whether miRNAs are preferentially loaded into exosomes for secretion or retention in cells are a key question that is still poorly understood. Some studies have shown that Argonaute 2 (AGO2) phosphorylation [38] and RNA-binding proteins such as hnRNPA2b [39] and Y-box protein 1 [40] may alter ncRNAs sorting and loading as well as release by exosomes. These packaged “harmful” ncRNAs disrupt the microenvironment of tissue sites and even distant organs and promote the acquisition of pre-metastatic niches. They may be one of the most powerful tumor substitutes.

## 3. Cancers Caused by Pathogens

Pathogenic microorganism infections are responsible for up to 20% of cancer cases worldwide [41,42]. The recognition of persistent pathogen stimuli promotes a pronounced inflammatory response. For example, recognizing PAMPs through Toll-like receptors (TLR) triggers the innate inflammatory response. It is believed that the transfer and adhesion of microorganisms or the long-distance release of microbial metabolites activated by inflammation may promote various cancers. These microorganisms or their products can spread to the metastatic site with tumor cells and become the source of inflammation during metastasis [43]. Many studies have suggested that the interaction of pathogenic microorganisms with ncRNAs is involved in the occurrence and development of inflammation-related cancers.

### 3.1. Hepatitis B Virus-Induced Hepatocellular Carcinoma

**Figure 3:**
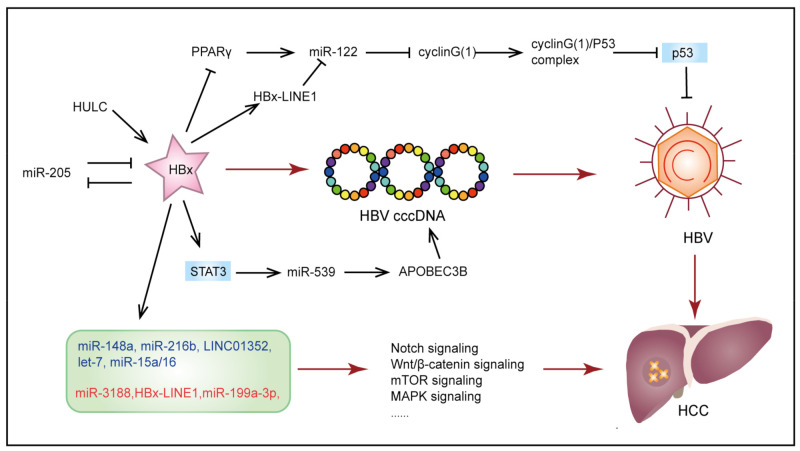
A schematic diagram of the role of ncRNAs in HBV-induced hepatocellular carcinoma. A negative feedback loop is formed between miR-205 and HBx. HULC/HBx/STAT3/miR-539/APOBEC3B signaling is involved in enhancing the stability of HBV cccDNA, thereby promoting HBV replication. The dysregulation of miR-122 is involved in eliminating p53-mediated inhibition of HBV replication. The ncRNAs in the light green rounded rectangle are believed to be dysregulated by HBx. Among them, the expression of ncRNAs in dark blue is inhibited by HBx, while the expression of ncRNAs in red is promoted. Dysregulated ncRNAs participate in the activation of oncogenic pathways, thereby promoting the occurrence and development of HCC.

Globally more than 50% of HCC cases are related to HBV infection that induces chronic necroinflammation [44,45]. Soon after infection, HBV DNA is converted into covalently closed circular DNA (cccDNA), a template responsible for transcribing all viral mRNAs and persistent HBV infection [46,47]. Then, the HBV X protein (HBx) is recruited to the cccDNA minichromosomes in HBV replicating cells to increase cccDNA transcription and viral replication [48,49]. Various ncRNAs have been reported to be involved in HBV replication and the initiation and development of HCC (Figure 3).

A previous study used ChIP-Seq on HBx-bound fragments in a cccDNA-driven HBV replication system and found that HBx specifically binds to a large number of target sequences, including protein-coding genes and ncRNAs (16 lncRNA promoters and 32 lncRNAs intragenic regions, 44 snoRNAs, and 75 miRNA promoter regions) [50]. HBx and ncRNA networks may activate HBV replication and affect the viral load in hepatic necrosis inflammation, thus contributing to carcinogenesis. For example, HBx epigenetically inhibits tumor suppressor miR-205 by inducing promoter hypermethylation. HBx mRNA is also a direct target of miR-205. The epigenetic silence of miR-205, in turn, up-regulates HBx [51]. Long ncRNA HULC has also been proven to maintain the stability of HBV cccDNA and activate HBV replication by modulating HBx/STAT3/miR-539/APOBEC3B signaling [52]. Non-coding RNAs can also be negatively regulated by HBx. For example, miR-122 is the most highly expressed miRNA in the liver, but it is found that miR-122 expression in the liver of HBV-infected patients is significantly down-regulated. It has been reported that HBx down-regulates miR-122 by binding and inhibiting peroxisome proliferator activated receptor-gamma (PPARγ) which is able to recognize miR-122 DR1 and DR2 motifs to regulate its transcription [53]. Furthermore, Liang et al. found that HBx-LINE1 acted as a molecular sponge to reduce the expression of miR-122, thereby promoting hepatocyte EMT-like changes [54]. In turn, loss of miR-122 is reported to lead to up-regulation of cyclin G(1) (miR-122 direct target), which initiates the formation of cyclin G(1)-p53 complex, thereby eliminating p53-mediated inhibition of HBV replication [55].

Non-coding RNAs dysregulated by HBx have a key role in promoting HBV-related HCC initiation and development. Recent research demonstrated that TGF-β1 and HBx cooperate to promote the malignant transformation of hepatic progenitor cells through a JNK/c-Jun/miR-199a-3p dependent pathway [56]. Tumor suppressor let-7 negatively regulates cell proliferation by targeting STAT3. Tumor repressive miR-15a/16 acts as a key regulator of cell cycle checkpoints from the G1 phase to the S phase by targeting cyclin D1. It is found that HBx down-regulated both of them, and repression of these tumor suppressors is required for the HBx-induced hepatocarcinogenesis [57,58]. The potential regulation between HBx and ncRNAs is also involved in the regulation of many cancer-related signal pathways. For example, HBx inhibits the activation of miR-148a [59] and miR-216b [60] by mediating p53, thus promoting the activation of hematopoietic pre-B cell leukemia transcription factor-interacting protein (HPIP) and insulin-like growth factor 2 mRNA-binding protein 2 (IGF2BP2) and activating AKT/mTOR and MAPK/ERK signaling pathways, respectively. HBx also promotes cAMP-response element binding protein (CREB)-mediated activation miR-3188, thereby inhibiting Zinc Fingers And Homeoboxes 2 (ZHX2) and activating the Notch signal [61]. Moreover, Wnt/β-catenin signaling activation is also associated with HBx-LINE1 [33] and HBx/estrogen receptor (ERα)/LINC01352/miR-135b/adenomatous polyposis coli (APC) axis [62].

### 3.2. Human Papillomavirus-Induced Cervical Cancer

It is believed that high-risk (HR) human papillomavirus (HPV) infection is the main cause of the occurrence of cervical cancer (CC) [63]. In the advanced stage of CC, precancerous lesions and invasive CC are usually related to strong inflammatory infiltration such as macrophages, T cells, Th17 cells, dendritic cells, and monocytes in the stroma [64,65,66,67]. 

HPV-16 and HPV-18 are the most common virus types [68]. HPV gene products can be additionally divided into core protein and accessory protein. It has commonly been assumed that E6 and E7 genes, which encode accessory proteins, are key factors contributing to the carcinogenicity of HPV [69]. Specifically, E6 and E7 oncoproteins inactivate antiproliferative p53 and pRb tumor-suppressing pathways, respectively, thereby inducing unplanned cell cycle progression [69,70,71]. Non-coding RNAs may participate in the regulation of HPV E6/E7 gene expression, thereby regulating the expression of HPV oncoproteins. Sannigrahi et al. used computer software to screen out miRNAs that have binding sites in HPV-16 mRNA. The best candidate miRNA is miR-139-3p, and it is significantly down-regulated through promoter methylation in HPV-positive tissues and cells. The inhibition of miR-139-3p increases the expression of E6/E7 and promotes cell proliferation and migration, and induces resistance to cisplatin and 5-fluorouracil of CC and head and neck cancer (HNC) [72]. Furthermore, miR-375 inhibits HR HPV E6 and E7 and directly targets E6-associated protein (E6AP) [73], which is known for interacting with E6 as well as targeting p53 and causing the degradation of its protein [74]. Long ncRNA TMPOP2 is another good example of crosstalk between E6 and E7 viral proteins and ncRNAs. The degradation of p53 induced by E6 and E7 relieved its inhibition on TMPOP2. High expression of TMPOP2, in turn, isolates miR-375 and miR-139, causing increased expression of E6/E7 proteins [75].

HPVs are small, double-stranded DNA viruses that enable to encode ncRNAs to promote their life cycle and CC carcinogenesis. For example, 9 HPV-encoded miRNAs have been identified in human cervical lesions and HPV-16 transfected cell lines. HPV-16-miR-H1-1 and HPV-16-miR-H2-1 seem to participate in cell cycle progression, migration, and immune response and are essential for virus infection and maintenance [76]. Furthermore, a recent study found that HPV-derived circE7 can be translated to E7 oncoprotein and may promote virus replication and host cell transformation [77]. The established stability and low translation activity of circRNAs may be particularly suitable for promoting the adaptability of infected cells during the incubation period. Therefore, this may also partly explain how HR HPVs progress from latent infections to incurable cancers. 

In addition, a large amount of data supports the role of E6 and E7 in regulating miRNA networks, and most of these miRNA disorders are related to carcinogenesis. For example, in HPV-16-positive and HPV-18-positive CC cell lines, the E6 protein down-regulates the tumor suppressor miR-34a [78] and miR-23b [79] by inhibiting p53, thereby leading to cell cycle progression and increased expression of urokinase-type plasminogen activator (uPA), respectively. Similarly, the HR HPV E7 gene induces pRb degradation, thus releasing E2F, which eventually leads to the up-regulation of carcinogenic miR-182 through the TGF-β/Smad signaling pathway [80]. E6 and E7 also down-regulate miR-146a-5p and up-regulate the expression level of lncRNA SNHG12 through c-MYC. Inhibited miR-146a-5p up-regulates its target histone lysine demethylase 2B (KDM2B) to promote the proliferation of CC cells [81], and SNHG12 promotes EMT through the ERK/Slug/E-cadherin pathway [82].

### 3.3. Helicobacter pylori-Induced Gastric Cancer

*H. pylori* is the most common pathogen found in the stomach [83]. It has been regarded as Class I or an exact carcinogen with the ability to alter host physiology and subvert the host immune response [84]. As discussed above, *H. pylori* infection induces abnormal DNA methylation in the gastric mucosa. Epigenetically silenced ncRNAs such as miR-210, miR-490-3p and miR-124 are involved in multiple steps in developing GC. Hypermethylation-mediated silencing of miR-490-3p reactivates SWI/SNF-related, matrix-associated, actin-dependent regulator of chromatin, subfamily d, member 1 (SMARCD1), a chromatin remodeling complex subunit, to impart a malignant phenotype of GC cells [85]. Furthermore, down-regulation of miR490-3p also promotes gefitinib resistance by inducing activation of DARPP-32/PI3K/Akt and STAT3 signaling pathways [86]. Inhibition of miR-210 expression enhances cell proliferation by activating its target stathmin 1 (STMN1) and dimethyladenosine transferase 1 (DIMT1) [87]. As for miR-124, suppressed miR-124 negatively regulates its target gene, Spermine oxidase (SMOX), which leads to DNA damage and subsequent tumorigenesis [88].

The pathogenicity of *H. pylori* is mainly attributed to its various virulence components. Among them, the cytotoxin-associated gene A (CagA) protein is the most important and the most widely studied. After reaching the epithelial cells, *H. pylori* uses T4SS to inject CagA into the host epithelial cells and immune cells, changing the host cell signal transduction and increasing the risk of GC [89,90]. However, it has been found that CagA is not necessary for the maintenance of malignant phenotypes in established GC cells. Therefore, subsequent genomic and epigenomic alterations are required to compensate for the “hit-and-run” process of CagA-induced gastric carcinogenesis [91]. Non-coding RNAs are possible links in the process of CagA-directed cancer. For example, miRNA microarray verified that *H. pylori* induced miR-223-3p expression in a CagA-dependent manner, and increased miR-223-3p directly targeted AT-rich interacting domain containing protein 1A (ARID1A) to regulate p21 and E-cadherin and eventually promote gastric carcinogenesis [92]. A recent study also reported that *H. pylori* infection changed the DNA repair system through the SNHG17/NONO and SNHG17/miR-3909/ RING1/Rad51 pathways. The upregulation of SNHG17 by CagA may partly explain the process of gastric carcinogenesis from *H. pylori* infection [93].

### 3.4. Cancers Associated with Other Pathogens

#### 3.4.1. Intestinal Flora-Induced Colorectal Cancer

The interaction between the host and the intestinal flora is essential for shaping the homeostasis of the intestine. Using the same colon tumor induction protocol, mice and rats that were reared under conditions involving the colonization by conventional intestinal flora suffered a higher tumor burden compared with aseptic conditions [94,95]. Certain members of the microbiota may modulate local immune responses and contribute to the development of a pro-inflammatory environment and CRC [94,96]. Non-coding RNAs may be important mediators of the host-intestinal flora communication network. For example, studies have confirmed that the nucleus of *Fusobacterium nucleatum* (*F. nucleatum*), reported to promote CRC [97]) activates TLR4-MyD88 innate immune signaling and causes the down-regulation of miR-18a* and miR-4802, which in turn increases their target genes Unc-51 Like Autophagy Activating Kinase 1 (ULK1) and Autophagy Related 7 (ATG7), two key components of the autophagy pathway, thereby regulating CRC chemoresistance [98]. In addition to the obvious influence of the intestinal microbiota on the expression of miRNAs and their target genes in CRC, studies have shown that host-derived miRNAs may also affect the activity of intestinal flora and promote the development of CRC. For example, miR-515-5p and miR-1226-5p, which are abundant in host stool samples, enter Escherichia coli and *F. nucleatum* to regulate bacterial gene transcription and co-localize with bacterial nucleic acid, thereby promoting transcription of the bacterial gene and the growth of bacteria [99].

#### 3.4.2. Hepatitis C Virus-Induced Hepatocellular Carcinoma

Patients with Hepatitis C virus (HCV) infection are at a high risk of developing cirrhosis and HCC [100]. Unlike HBV, HCV is a positive-strand RNA virus that does not integrate into the host genome [101]. The complex interplay between HCV and host ncRNAs has been gradually revealed, and it has been found that ncRNAs are deeply involved in HCV replication and subsequent carcinogenesis. For example, miR-122 stabilizes the HCV RNA and inhibits its degradation by directly targeting genomic RNA 5′ UTR [102,103]. HCV also inhibits miR-122 targets by sequestering miR-122 from its endogenous mRNA targets [104]. Furthermore, miR-141 is required for HCV replication by inhibiting tumor suppressor gene DLC-1 (a Rho GTPase-activating protein) [105]. In addition, overexpression of miR-199a-3p inhibited HCV replication by directly targeting the internal ribosomal entry site of the HCV genome [106]. On the other hand, increased miR-155 induced by HCV activated Wnt signaling in vitro and in vivo to promote hepatocyte proliferation and tumorigenesis [107].

## 4. Cancers Associated with Environmental Factors

### 4.1. Inhalation Exposure-Induced Cancer

#### 4.1.1. Cigarette Smoke

CS has a strong carcinogenic effect on multiple organs, leading to increased morbidity and mortality globally [108,109]. CS is a complex mixture containing more than 4500 compounds including carbon monoxide, ammonia, acrolein, acetone, nicotine, benzopyrenes, hydroquinone, and nitrogen oxides. Long-term exposure to CS promotes inflammation and immunological changes in the lung and the whole body [110]. It has been shown that the function of immune cells is modulated by CS. For example, NK cells, a lymphoid cell type, are reduced in smokers compared with non-smokers, which may cause an increased risk of various cancers [111]. In addition, nicotine induces neutrophils to release neutrophil extracellular traps (NETs) in a dose-dependent manner. NETs serve to respond to large pathogens [112], while aberrant NETs release contributes to tissue damage and excessive inflammation [113]. Additionally, nicotine is the main addictive ingredient in tobacco products. Tobacco addiction may be the cause of CS-mediated pathogenesis. It has been proven that miR-504 increases the expression of the dopamine D1 receptor gene (DRD1), which is related to nicotine dependence [114]. A number of studies suggested ncRNAs function in CS-induced cancer initiation and progression. 

#### 4.1.2. Lung Cancer

Lung cancer (LC) is the leading cause of cancer deaths worldwide. The vast majority of LC patients are active smokers or have a long history of smoking. Emerging evidence suggests CS promotes LC progression by mediating the abnormal expression or activity of epigenetic regulators [115,116,117]. Alterations in DNA methylation may be one potential mechanism mediating CS-induced changes in gene profiles [118]. It has been reported that CS suppresses gene expression by upregulating DNMT1, furthermore, the inhibition of DNMT1 restores the expression of genes through demethylation [119]. The level of histone acetylation was also found to be related to CS exposure. Active smoking may promote acetylation of histone H4, while ex-smokers showed increased histone H3 acetylation [15]. Epigenetic modification mechanisms can be a reasonable explanation of the association between CS exposure and gene expression change. Eighty-two percent of miRNAs expression showed a downward trend in smokers. Specifically, tumor-suppressive miR-200 family (miR-200b, miR-200c), miR-205, and miR-487b are epigenetically silenced under tobacco carcinogens exposure [120,121], which leads to malignant transformation of human bronchial epithelial cells (HBECs) and the malignant phenotype of LC cells through promoting EMT and reactivating the Wnt pathway.

There is growing evidence that KRAS mutations in LC are closely related to tobacco exposure history. Moreover, KRAS mutations are also mutually exclusive with EGFR mutations that are more common in non-smoking LC [122,123,124]. Non-coding RNAs may be a new regulatory pathway for KRAS-driven LC. Chin et al. found a variant allele in the let-7 complementary site of KRAS 3’UTR, which changed let-7-mediated KRAS expression, and was associated with the increased risk of non-small cell lung cancer (NSCLC) in moderate smokers [125]. Moreover, miR-193a-3p was found to directly target KRAS [126]. In addition, the miR181ab1 cluster (miR181a1 and miR181b1) is a key regulator of KRAS-driven carcinogenesis, including LC and pancreatic ductal adenocarcinoma (PDAC) [127]. By targeting tyrosine kinase FAK and laminin subunit LAMB3, miR-1298 inhibits the growth of KRAS-driven cancer cells [128]. At present, few therapies are available for patients with KRAS-driven cancers. Nanoliposome packaged miR-193a-3p has shown broad applicability as a therapeutic agent to target KRAS-mutant cancer [126]. Further research of these miRNAs may help determine the best chemotherapy regimen.

#### 4.1.3. Esophageal Cancer

Previous studies have suggested an association between esophageal cancer (EC) and smoking [129]. Increasing evidence has also proved the relationship between inflammation-related biomarkers and the risk of EC [130,131]. A prospective study provided evidence that systemic inflammatory status has an important role in the etiology of esophageal adenocarcinoma (EAC). Smoking as an inflammatory exposure is also emphasized in mediating the inflammatory mechanism in the development of EC to a certain extent [132]. Non-coding RNAs may have an important role in the tobacco-induced initiation and development of EC. For example, epigenetic repression of miR-217 mediated by cigarette smoke condensate (CSC) contributes to the pathogenesis of EAC via upregulation of kallikrein 7 (KLK7) [133]. Additionally, according to epidemiological evidence, the incidence of esophageal squamous cell carcinoma (ESCC) has a clear gender preference. The incidence of ESCC in men is two to four times that of women, and this gender difference is partly due to male-specific factors such as smoking and sex hormones [134,135,136]. Such gender bias might be related to the Yin Yang 1 (YY1) blocking micropeptide (YY1BM) encoded by the Y chromosome-linked lncRNA LINC00278. Specifically, smoking exposure decreased m6A modification of LINC00278 and YY1BM translation. The low expression of YY1BM increased expression of eEF2K and inhibited apoptosis, thus conferring ESCC cells more adaptive to nutrient deprivation [137]. Based on these findings, LINC00278 may be a potential anti-cancer factor for male ESCC related to smoking.

#### 4.1.4. Pancreatic Cancer

Unlike other environmental factors, smoking is the first recognized adjustable risk factor for pancreatic cancer (PC) [138,139]. A dose–response relationship was found between the duration and intensity of smoking and the increased death rate and decreased survival rate of PC [140,141]. Non-coding RNAs may be involved in the connection between smoking and PC. Zhang et al. found that CSC promoted the excessive maturation of miR-25-3p by enhancing the m6A modification of miR-25-3p. Excessive miR-25-3p promotes the initiation and development of PC by targeting leucine-rich repeat protein phosphatase 2 (PHLPP2) and activating oncogenic AKT-p70S6K signaling [142].

### 4.2. Ingestion Exposure-Induced Cancers

Toxic metals are widely present in the environment, contaminating food and drinking water. Among the many metal contaminants that humans are frequently exposed to, arsenic, cadmium, nickel, and hexavalent chromium, are classified as Class I carcinogens by the International Agency for Research on Cancer (IARC). The toxic mechanism of heavy metals is classically considered to promote redox disorders as well as the production of inflammatory mediators and change the function of mitochondria [143]. Recently, with cadmium and arsenic as the main mediator, there is growing evidence that toxic metals may exert their toxicity through non-coding molecules, and ncRNAs may be novel biomarkers and key modulators of toxicological reactions in toxic metal-induced cancers.

#### 4.2.1. Arsenic

Arsenic is a well-known human carcinogen in the natural environment. Arsenic in drinking water is one of the most serious environmental health threats worldwide. Long-term intake of arsenic increases the risk of tumors in the skin, bladder, liver, kidney, lung, and other tissues [144]. Unlike other classical environmental carcinogens, arsenic is not so able to induce gene mutations at relevant exposure concentrations. Several studies have explained the genotoxicity of arsenic, such as inducing micronuclei (MN), DNA strand breaks, sister chromatid exchanges (SCE), CA, and aneuploidy [145]. Epigenetic mechanisms such as DNA methylation and histone modification can also be included in extensive changes in global gene expression in individuals after arsenic exposure [146,147,148]. Various molecular mechanisms have been proposed to explain arsenic-induced carcinogenesis, among which arsenite-induced inflammation mediated by HIF-2α may be related to malignant transformation of cells [149]. Studies have shown that the dysregulation of ncRNAs derived from exosomes under arsenic exposure may be an important link in the process from arsenite-induced inflammation to malignant transformation. Specifically, in arsenite-transformed human liver epithelial cells (L-02), NF-κB activation promotes up-regulation of exosome-derived miR-155. Exosomes transfer miR-155 to surrounding cells, thereby inducing pro-inflammatory activity of normal liver cells [150]. The up-regulation of miR-155 promotes the acquisition of arsenic-induced cancer stem cell-like characteristics by inhibiting the expression of KH domain containing RNA binding (QKI) [151]. In addition, the increased circRNA_100284 transferred by exosomes from transformed L-02 cells has also been proven to promote an accelerated cell cycle and proliferation of normal liver cells via miRNA-217 regulation of enhancer of zeste homolog 2 (EZH2), which may be involved in the malignant transformation of human hepatic cells induced by arsenite [152].

The association from inflammation to EMT in arsenic-induced malignant transformation of immortalized human keratinocytes (HaCaT cells) [153] and HBECs [154,155] may be defined by miR-21. It is demonstrated that under arsenic exposure, secretion of IL-6 acts on the activation of STAT3, thus increasing miR-21 [153,155]. Up-regulated miR-21 targets Programmed Cell Death 4 (PDCD4) which interacts with Twist Family BHLH Transcription Factor 1 (Twist1) and inhibits its expression and function, thus contributing to the EMT [154]. On the other hand, arsenite-induced angiogenesis [156] and malignant transformation of normal cells [157] are also found to be related to miR-21.

The miR-200 family may also have a causal role in arsenite-induced cell migration. Numerous studies have reported that the miR-200 family inhibits EMT and cancer metastasis. Zinc-finger E-box-binding homeobox factor 1 (ZEB1) and ZEB2, the most critical conversion molecules of EMT, inhibit the miR-200 family and are also targets of miR-200, forming a double-negative feedback loop [158,159,160]. Further mechanistic studies revealed that miR-200b inhibits arsenic-transformed cell migration by targeting protein kinase Cα (PKCα) and Wnt5b-PKCα positive feedback loops and subsequently inhibiting Rac Family Small GTPase 1 (Rac1) activation [161].

#### 4.2.2. Cadmium

Cadmium is a common environmental pollutant, and the main source of human exposure occurs through cadmium-contaminated food, water, and air. It is reported that cadmium induces DNA damage and chromosomal aberrations [162]. However, cellular mutations are not likely to occur, because that genotoxicity and mutagenicity induced by cadmium increases apoptosis in 50% of exposed cells [163]. Additionally, aberrant DNA methylation is also a possible mechanism through which cadmium triggers carcinogenesis. In adolescent mouse models, continuous biological accumulation of cadmium activates NLRP3 inflammasomes and up-regulates pro-inflammatory cytokines (IL-1α, IL-1β, and IL-18) to induce oxidative stress and inflammatory responses, leading to toxic responses [164]. Many studies have shown that the dysregulation of ncRNAs during the toxicological reaction to cadmium may be an important step in cadmium carcinogenesis.

The miR-221 family is highly expressed and function as oncogenes in various cancers [165,166]. A cross-sectional study of people with occupational cadmium exposure showed that miR-221 in the cadmium-exposed group was significantly up-regulated compared with the unexposed group. Serum levels of pro-inflammatory cytokines IL-6, TNF-α, IL-17, and Th17 were positively correlated with the expression of miR-221 in the exposed group [167]. In addition, down-regulation of miR-30 induced by cadmium alleviates its inhibition on Snail, an EMT master regulator in lung epithelial cells, thereby inducing EMT, which may be the molecular basis of cadmium-induced diseases [168].

In addition to miRNAs, lncRNAs have also been found to be regulated by cadmium. It has been shown that cadmium rapidly increased lncRNA GABPB1-AS1 and LINC00152 in human-induced pluripotent stem cells, while the protein-coding genes associated with pluripotency were only minimally regulated [169]. Another study reported that cadmium increased the expression of ENST00000414355 in the lungs of rats in a dose-dependent manner. What’s more, the expression of ENST00000414355 is positively correlated with the expression of genes related to DNA damage [170]. These data indicate the potential of using ncRNAs as early biomarkers and therapeutic targets for toxic cadmium exposure, but more research to reveal the underlying molecular mechanism of involvement of ncRNAs in cadmium-induced carcinogenesis is needed.

### 4.3. Dermal Contact Exposure-Induced Cancers

#### Ultraviolet

A long exposure to ultraviolet (UV) radiation from sunlight may be the most im-portant and inevitable carcinogen. UV can be subdivided into three wavebands: UVC (100~290 nm), UVB (290~320 nm), and UVA (320~400 nm). UVC rays are mostly fil-tered by atmospheric ozone, while UVB and UVA that can reach the earth’s surface are the main environmental risk factors that cause skin cancers [171,172], including non-melanoma skin cancer, squamous cell carcinoma (SCC), basal cell carcinoma (BCC), and melanoma. UV is known to induce chromatin remodeling, which is one of the hallmarks of epigenetic modification of the chromatin in model organisms. In ad-dition, the UVB region of the spectrum causes mutation through DNA base crosslink-ing and error-prone repair synthesis. DNA damage induced by cumulative exposure to UV in epidermal keratinocytes triggers a stress response, activation of p53, and DNA repair [173]. Typically, DNA base pair changes due to UV-induced mutation leads to changes in expression of tumor-related genes, which triggers abnormal cellular signal transduction [174]. Experiments in mice further proved that UV-induced neutrophilic inflammatory response promoted reciprocal melanoma–endothelial cell interactions, thus increasing the likelihood of intravasation and hematogenous dissemination [175]. UV-induced skin inflammatory responses are involved in the proliferation and migra-tion of melanocytes [176], and ncRNAs are likely to be one of the cellular mechanisms that link UV-induced inflammation with skin cancers. Whole transcriptome sequenc-ing showed that UVB irradiation of keratinocytes induced changes in the dou-ble-stranded domains of certain ncRNAs. These ncRNAs, especially snRNAs (the most abundant ncRNA present in UV-irradiated cells), induce non-radiation cells to pro-duce pro-inflammatory cytokines TNF-α and IL-6 through TLR3 mediation, thereby helping UVB induce skin inflammation [177]. Degueurce’s research identified passen-ger miR-21-3p as a pro-inflammatory miRNA that was activated by UV light through a PPARβ/δ and TGFβ-dependent manner in keratinocytes. High-level miR-21-3p ex-pression prevents excessive SMAD7 (miR-21-3p direct target) protein upregulation, while knockdown of miR-21-3p reduces the inflammation caused by UV, suggesting suppression of miR-21-3p a therapeutic benefit for the prevention of UV-induced skin inflammation [178]. In addition, another study confirmed that the high expression of miR-21-3p was related to SCC; miR-21-3p regulates the Rb/E2F cell-cycle axis by di-rectly targeting Phosphatase and Actin Regulator 4 (PHACTR4). More importantly, the regulation of PHACTR4 is miR-21-3p specific and is not observed with guide strand miR-21-5p [179]. Interestingly, miR-21-5p and miR-21-3p have dual but independent carcinogenicity, and both are related to UV-induced cancers; miR-21-5p has been proven to be an “oncomiR” that is related to UV induction [180] and promotes SCC formation by targeting tumor suppressors grainyhead-like transcription factor 3 (GRHL3) and phosphatase and tensin homolog deleted on chromosome ten (PTEN) [181]. On the other hand, the expression, activity, and function of lncRNAs have also been found to be affected by UV and may promote the development of skin cancer. For example, in mouse and human keratinocytes, lincRNA-p21 is induced in a p53-dependent pathway in response to UVB stimulation and is involved in triggering UVB-induced apoptotic death [182]. However, a single p53 allele is often mutated or deleted as an early carcinogenic event during the development of early skin cancer [183]. The mutation of a single p53 allele inhibits the inducing of lincRNA-p21 expres-sion by UVB and subsequent UVB-induced apoptosis.

## 5. ncRNAs as Therapeutic Targets

As listed in Table 1 and Table 2, expressions of ncRNAs are abnormally regulated in different inflammation-related cancers and their expression levels are associated with tumor occurrence and development, making them potential targets for inflammation-related cancer therapy. The potential and challenges of their therapeutic exploitation have aroused a lot of interest worldwide [184]. Chemical modification technology and nanotechnology have continuously improved the in vivo biological activity of oligonucleotides and cancer cell-specific delivery, which might be the unique advantage of ncRNA as a treatment. Developing therapeutic interventions based on virus-encoded ncRNAs has shown prospective promise. One of the most exciting findings in the world of viruses and ncRNAs is miR-122, a virus-encoded miRNA, which is essential for HCV replication. Strategies to reduce the HCV titer in patients through functional isolation of miRNA-122 by using the anti-microRNA drug Miravirsen (SPC3649) have already been clinically viable [185]. Additionally, synthetic miRNA sponges containing repeated miRNA antisense sequences act as specific miRNA inhibitors by preventing them from binding to endogenous targets [186]. The first artificial circRNA that acts as a miR-122 sponge has been reported to have the same efficacy as the Miravirsen [187]. As for ncRNAs transcriptionally regulated by inflammation stimuli, inhibition of TFs binding to respective promoters of ncRNAs to alter ncRNA promoter activity is a possible way to modulate their expression. Exosomes have been increasingly studied as drug delivery tools due to their stability, biocompatibility, and low immunogenicity. It has been experimentally confirmed that exogenous miRNAs can also be sorted into exosomes [188,189]. As discussed, inflammation stimuli can exploit exosomes as delivery vectors to transfer ncRNAs to other non-infected cells, thus changing the expression of tumor-suppressive or tumor-promoting ncRNAs. Hence, exogenous ncRNAs or RNA interference (RNAi) transferred by exosomes may be a promising method for gene therapy. This has been supported by several pieces of research on protein-coding genes, such as silencing MAPK [190] and RAD51 [191] by combining exosomes with RNAi technology. Additional research is required to apply these therapies for ncRNAs and eventually develop them for clinical use. Moreover, recent studies have found that a small number of small open reading frames (sORFs) in ncRNAs have the potential to encode peptides or proteins. Specific ncRNAs and their encoding proteins or peptides can promote or inhibit inflammation-related cancers, which may help develop new anti-cancer therapeutic targets and cancer markers for diagnosis and prognosis. 

## 6. Conclusions and Perspectives

It has been gradually revealed that ncRNAs are an important regulatory layer of gene expression and signaling pathways in inflammatory conditions. Under inflammatory stimulation, some cancer-related ncRNAs are abnormally regulated by various mechanisms, including mediated by several inflammation-related TFs, abnormal epigenetic modifications, and exosomes delivery. Some exogenous ncRNAs derived from viruses may also be involved in the carcinogenesis process of inflammation. Uncontrollable ncRNAs further regulate different cancer-related processes (such as proliferation, metastasis, metabolism, and drug resistance), while some ncRNAs, in turn, promote inflammation (activation of inflammation-related TFs or promotion of encoding a microbial oncogenic protein, etc.), thus forming a positive feedback loop to increase inflammation stimulation and activate inflammation signal pathways continuously. In particular, exploring the use of ncRNA markers to predict the early stages of inflammation-related tumors and guide personalized drug treatment decisions also has broad prospects. For example, environmental stimulus exposure such as smoke is mutagenic, with somatic mutations (for example, KRAS) progressively acquired in epithelial cells after chronic smoke exposure, and inflammation and cancer risks such as LC and PC gradually increase. The treatment of LC is surgery for early stages, chemotherapy with concurrent radiation for some locally advanced cancers, and palliative chemotherapy for metastatic disease. However, inefficient methods of early diagnosis coupled with acquired drug resistance render LC a major clinical concern. In addition, PC has the highest fatality of any cancer type due to its aggressive nature and the lack of biomarkers. Tobacco usage is the only environmental factor agreed to confer a risk for PC. Gemcitabine has been the cornerstone of PC since its introduction in the mid-1990s, while the disease fails to be under control within weeks due to drug resistance [192]. The recognition of the alterations in ncRNAs provides a better understanding of the molecular basis for CS-medicated inflammation and carcinogenesis. Non-coding RNAs are well-known modulators of mechanisms implicated in drug resistance such as cell cycle control, apoptosis, and DNA damage repair, suggesting that ncRNA-based therapies could be a beneficial line of treatment, especially for the resistant phenotypes. Additionally, they have also been attributed to immune checkpoint regulation through controlling PD-1 and PD-L1 expression [193]. Additionally, mounting evidence supports the role of the gut microbiome in gastrointestinal cancer. *H. pylori*-induced GC is a multi-step comprehensive disease; thus, it is important to find screening strategies for detecting early-stage GC. Non-coding RNAs are promising clinical diagnostic biomarkers in GC, however, the most significant obstacles are the lack of ncRNAs with specificity and sensitivity. Furthermore, clinical techniques for detection of ncRNAs in plasma, serum, gastric juice, and urine are another important issue to be settled. Comprehensive knowledge of ncRNAs is needed before employing them as classification signatures of GC subtypes including EBV-positive, microsatellite instability, chromosomal instability, and genomic stable [194]. With the persistence of foreign antigens, virus-associated cancers, such as HCC and CC, are ideal candidates for immunotherapy compared to nonviral cancers [195]. For instance, E6 and E7 proteins of high-risk HPVs could function as foreign antigens and are perhaps the most common antigen targets for HPV therapeutic vaccines [196]. However, Kitamura et al. recently reported that E7 expression at mRNA and protein level is different in HPV16-positive HNC cell lines and clinical specimens, suggesting a potential mechanism that regulates the translation efficiency from viral mRNA [197]. E6 and E7 may even likely function as ncRNAs. Hence, since limited efficacy has been shown from therapeutic vaccines against HPV-related cancers, more research and clinical trials are needed. Additionally, many HCC-related clinicopathological parameters including HBV/HCV status, survival, recurrence, and metastasis formation have been linked to ncRNAs. Non-coding RNAs regulate cell homeostasis and promote HCC malignant phenotypes via controlling several intracellular pathways and interacting complex networks. Non-coding RNAs also show potential in HCC treatment. Clinical trials may provide the translational evidence to identify relevant ncRNAs for clinical implication. Of course, challenges remain and need to be overcome. There is still a lack of reliable in vivo models for the transition between inflammation and cancer. Furthermore, ncRNAs can regulate multiple targets at the same time and sometimes may participate in complex feedback mechanisms. We expect that the massive and complex knowledge continuously discovered in this field can gradually solve these problems, allowing us to unravel the complex crosstalk between inflammation, ncRNA, and cancer, so as to promote the development of ncRNA-based diagnostic tests and therapeutic interventions to benefit patients.

## Figures and Tables

**Table 1:** Overview of miRNAs in cancers regulated by inflammation.

miRNA	Expression	Target	Related Cancers	Related Inflammation	Reference
miR-205	↓ ^18^	HBx mRNA	HCC ^1^	HBV ^11^	[51]
miR-205	↓	unknown	LC ^2^	CS ^12^	[120]
miR-122	↓	cyclin G(1)	HCC	HBV	[53,54,55]
miR-122	↓	HCV mRNA	HCC	HCV ^13^	[102,103,104]
miR-199a-3p	↑ ^19^	unknown	HCC	HBV	[56]
miR-199a-3p	↓	HCV mRNA	unknown	HCV	[106]
let-7	↓	STAT3	HCC	HBV	[58]
let7	↓	KRAS	LC	CS	[125]
miR-15a/16	↓	cyclin D1	HCC	HBV	[57]
miR-148a	↓	HPIP	HCC	HBV	[59]
miR-216b	↓	IGF2BP2	HCC	HBV	[60]
miR-3188	↑	ZHX2	HCC	HBV	[61]
miR-139-3p	↓	E6/E7	CC ^3^, HNC ^4^	HPV ^14^	[72]
miR-375	↓	E6AP	CC	HPV	[73]
miR-34a	↓	unknown	CC	HPV	[78]
miR-23b	↓	uPA	CC	HPV	[79]
miR-182	↑	unknown	CC	HPV	[80]
miR-146a-5p	↓	KDM2B	CC	HPV	[81]
miR-210	↓	STMN1, DIMT1	GC ^5^	*H. pylori* ^15^	[87]
miR-490-3p	↓	SMARCD1, DARPP-32	GC	*H. pylori*	[85,86]
miR-124	↓	SMOX	GC	*H. pylori*	[88]
miR-223-3p	↑	ARID1A	GC	*H. pylori*	[92]
miR-18a*	↓	ULK1	CRC ^6^	*F. nucleatum*	[98]
miR-4802	↓	ATG7	CRC	*F. nucleatum*	[98]
miR-515-5p	unknown	unknown	unknown	*Escherichia coli* and *F. nucleatum* ^16^	[99]
miR-1226-5p	unknown	unknown	unknown	*Escherichia coli* and *F. nucleatum*	[99]
miR-141	↑	DLC-1	unknown	HCV	[105]
miR-155	↑	unknown	HCC	HCV	[107]
miR-155	↑	QKI	unknown	Arsenic	[150,151]
miR-504	unknown	DRD1	unknown	CS	[114]
miR-200b, miR-200c	↓	unknown	LC	CS	[120]
miR-200b	↓	PKCα	unknown	Arsenic	[161]
miR-487b	↓	SUZ12, BMI1, WNT5A, MYC, and KRAS	LC	CS	[121]
miR-193a-3p	↓	KRAS	LC	CS	[126]
miR181ab1	↓	unknown	LC, PDAC ^7^	CS	[127]
miR-1298	↓	FAK, LAMB3	LC	CS	[128]
miR-217	↓	KLK7	EC ^8^	CS	[133]
miR-25-3p	↑	PHLPP2	PC ^9^	CS	[142]
miR-221	↑	unknown	unknown	Cadmium	[167]
miR-30	↓	Snail	unknown	Cadmium	[168]
miR-21	↑	PDCD4	LC	Arsenic	[153,154,155,156,157]
miR-21-3p	↑	SMAD7	unknown	UV ^17^	[178]
miR-21-3p	↑	**PHACTR4**	SCC ^10^	unknown	[179]
miR-21-5p	↑	GRHL3, PTEN	SCC	UV	[180,181]

^1^hepatocellular carcinoma, ^2^ lung cancer, ^3^ cervical cancer, ^4^ head and neck cancer, ^5^ gastric cancer, ^6^ colorectal cancer, ^7^ pancreatic ductal adenocarcinoma, ^8^ esophageal cancer, ^9^ pancreatic cancer, ^10^ squamous cell carcinoma, ^11^ Hepatitis B virus, ^12^ cigarette smoke, ^13^ Hepatitis C virus, ^14^ Human papillomavirus, ^15^ *Helicobacter pylori*, ^16^ *Fusobacterium nucleatum*, ^17^ ultraviolet, ^18^ downregulated, ^19^ upregulated.

**Table 2:** Overview of lncRNAs in cancers regulated by inflammation.

lncRNA	Expression	Target	Related Diseases	Related Inflammation	Reference
HULC	↑ ^10^	unknown	HCC ^1^	HBV ^5^	[52]
HBx-LINE1	↑	miR-122	HCC	HBV	[54]
LINC01352	↓ ^11^	miR-135b	HCC	HBV	[62]
TMPOP2	↑	miR-375, miR-139	CC ^2^	HPV ^6^	[75]
SNHG12	↑	unknown	CC	HPV	[82]
SNHG17	↑	NONO, miR-3909	GC ^3^	*H. pylori* ^7^	[93]
LINC00278	↓	unknown	EC ^4^	CS ^8^	[137]
GABPB1-AS1	↑	unknown	unknown	Cadmium	[169]
LINC00152	↑	unknown	unknown	Cadmium	[169]
ENST00000414355	↑	unknown	unknown	Cadmium	[170]
lincRNA-p21	↓	unknown	unknown	UV ^9^	[182]

^1^hepatocellular carcinoma, ^2^ cervical cancer, ^3^ gastric cancer, ^4^ esophageal cancer, ^5^ Hepatitis B virus, ^6^ Human papillomavirus, ^7^ *Helicobacter pylori*, ^8^ cigarette smoke, ^9^ ultraviolet, ^10^ upregulated, ^11^ downregulated.
